# Vitamin D and Hemopoietic Stem Cell Transplantation: Clinical Guidance for GVHD Management and Post-Transplant Outcomes

**DOI:** 10.3390/cancers18060972

**Published:** 2026-03-17

**Authors:** Manlio Fazio, Maria Elisa Nasso, Sebastiano Gangemi, Adele Bottaro, Luca Gammeri, Fabio Stagno, Alessandro Allegra

**Affiliations:** 1 Division of Hematology, Department of Human Pathology in Adulthood and Childhood “Gaetano Barresi”, University of Messina, Via Consolare Valeria, 98125 Messina, Italy; manlio.fazio@polime.it (M.F.); maria.nasso@studenti.unime.it (M.E.N.); adele.bottaro@studenti.unime.it (A.B.); fabio.stagno@unime.it (F.S.); aallegra@unime.it (A.A.); 2 Allergy and Clinical Immunology Unit, Department of Clinical and Experimental Medicine, University of Messina, Via Consolare Valeria, 98125 Messina, Italy; gangemis@unime.it; 3 Department of Biomedical and Dental Science and Morphofunctional Imaging, University of Messina, Via Consolare Valeria, 98125 Messina, Italy

**Keywords:** vitamin D, hematopoietic stem cell transplantation, immune synapses, graft-versus-host disease, engraftment, relapse, interventional strategies

## Abstract

Vitamin D is a pleiotropic secosteroid with endocrine and intracrine actions that influence key phases of allogeneic hematopoietic stem cell transplantation. Epithelial barriers, antigen-presenting cells and effector lymphocytes express the vitamin D receptor and enzymes required for local activation, allowing circulating 25-hydroxyvitamin D to be converted into its active form and modulate immune interactions. During the peri-transplant period, sunlight deprivation, reduced intake, mucosal injury, cholestasis and corticosteroid exposure markedly reduce vitamin D levels at a time when antigen presentation and immune reconstitution occur. This review integrates mechanistic immunology with clinical observations and interventional data to outline strategies that prevent severe deficiency. It summarizes epidemiology before and after transplantation, associations with acute and chronic graft-versus-host disease, relapse, engraftment, infections, bone health and survival, and evaluates dosing approaches including pre-conditioning loading and reassessment at day thirty with escalation if needed. Absorption-savvy formulations such as oral thin-film and intramuscular cholecalciferol are considered when gastrointestinal function is compromised. Given the high prevalence of deficiency, biological plausibility, safety and low cost, a structured approach that includes screening, repletion and monitoring to achieve concentrations of at least thirty nanograms per milliliter by day thirty represents a pragmatic and low-risk component of supportive care pending definitive evidence.

## Simple Summary

Vitamin D regulates key immune processes involved in allogeneic hematopoietic stem cell transplantation. Many immune and barrier cells can locally activate vitamin D, allowing it to modulate inflammation and immune reactivity. During the transplant period, factors such as limited sunlight exposure, reduced intake, mucosal injury, cholestasis and corticosteroid use frequently lead to low vitamin D levels, exactly when antigen presentation and immune recovery are most active. This review summarizes how vitamin D status changes before and after transplantation and how deficiency relates to graft-versus-host disease, relapse, infections, engraftment, bone health and survival. It also discusses strategies to prevent severe deficiency, including pre-conditioning supplementation, reassessment around day 30, and dose adjustment if needed. When gastrointestinal absorption is impaired, intramuscular or oral thin-film formulations may be useful. Given the high prevalence of vitamin D deficiency after hematopoietic stem cell transplantation and the generally favorable safety profile of physiologic supplementation, maintaining serum 25-hydroxyvitamin D concentrations ≥30 ng/mL by approximately day +30 may represent a pragmatic supportive strategy. However, it is important to emphasize that, to date, randomized clinical trials demonstrating a direct effect of vitamin D repletion on transplant outcomes such as graft-versus-host disease incidence, immune reconstitution, or survival remain limited. From a clinical perspective, the rationale for monitoring and correcting vitamin D deficiency in hematopoietic stem cell transplantation recipients primarily derives from indirect evidence linking low 25-hydroxyvitamin D levels with impaired immune recovery, increased infection risk, and skeletal complications in observational studies. Potential benefits therefore include support for bone health and maintenance of physiologic immunomodulatory pathways. The principal risks of supplementation at conventional doses are limited and mainly relate to hypercalcemia in susceptible individuals, particularly those with renal impairment, granulomatous disease, or dysregulated calcium metabolism, underscoring the importance of biochemical monitoring.

## 1. Introduction

### 1.1. General Considerations on the Relationship Between Vitamin D and Hematopoietic Stem Cell Transplantation

Allogeneic hematopoietic stem cell transplantation (allo-HSCT) embodies the tension between curative intent and iatrogenic injury [1]. Conditioning regimens damage epithelial and endothelial barriers, release damage-associated molecular patterns (DAMPs), and activate antigen-presenting cells (APCs) [2]. Donor T cells engage in amplified synapses within a cytokine-rich environment that can culminate in acute and chronic graft-versus-host disease (aGVHD and cGVHD) [3]. Over the last two decades, vitamin D biology has moved beyond skeletal homeostasis to a nuanced immunology wherein dendritic cells, macrophages and lymphocyte subsets express both the vitamin D receptor and the 1-alpha hydroxylase (CYP27B1) [4]. These cells can locally convert 25-hydroxyvitamin D [25(OH)D] into 1,25-dihydroxyvitamin D [calcitriol or 1,25(OH)_2_D] and thereby modulate co-stimulation, cytokine output, trafficking cues, and effector functions at the immune synapse [5]. The immune synapse refers to the highly organized interface between a T cell and an APC, characterized by spatially and temporally coordinated clusters of receptors, adhesion molecules, and signaling complexes that orchestrate antigen recognition and downstream activation [6].

In parallel, the clinical trajectory of HSCT reliably depresses 25(OH)D through inpatient confinement, anorexia and mucositis, cholestasis and diarrhea, corticosteroid-driven catabolism, and drug–nutrient interactions [7]. This convergence of mechanistic plausibility and predictable deficiency invites a pragmatic integration of vitamin D into supportive care without implying that it replaces established GVHD prophylaxis or that outcome benefits are already proven [8].

This narrative review was based on a targeted literature search designed to identify studies evaluating the role of vitamin D in the HSCT setting, with particular focus on GVHD, immune recovery, bone health, and post-transplant complications. Electronic databases including PubMed, Scopus, and Web of Science were searched for articles published between January 2000 and March 2025. Search terms included combinations of “vitamin D,” “25-hydroxyvitamin D,” “hematopoietic stem cell transplantation,” “HSCT,” “graft-versus-host disease,” “immune reconstitution,” and “bone mineral density.” Priority was given to prospective studies, randomized clinical trials, cohort studies, and systematic reviews involving adult or pediatric HSCT recipients. Additional relevant publications were identified through manual screening of reference lists from key articles. Preclinical and mechanistic studies were included when they provided insight into vitamin D-mediated immunomodulatory pathways relevant to transplantation biology. Articles not available in English or not directly related to HSCT outcomes were excluded.

### 1.2. Mechanistic Considerations

Vitamin D belongs to a group of fat-soluble secosteroids, characterized by a steroid backbone in which one of the rings is broken. The major circulating form used to assess vitamin D status is 25(OH)D, produced in the liver by hydroxylation of vitamin D_3_ (cholecalciferol) or vitamin D_2_ (ergocalciferol). Chemically, 25(OH)D retains the classical cholesterol-derived steroid framework with three intact rings and an opened B-ring, forming the characteristic secosteroid structure. A hydroxyl group at the C-25 position of the side chain distinguishes this metabolite from its precursor, vitamin D, and increases its polarity and binding affinity for vitamin D-binding protein in circulation. Although 25(OH)D itself has limited biological activity, it serves as the principal substrate for further hydroxylation by the enzyme CYP27B1 to generate the hormonally active metabolite 1,25(OH)_2_D, which exerts its effects through the vitamin D receptor in multiple tissues [9].

Vitamin D metabolism operates through two interconnected but functionally distinct pathways: the classical endocrine axis and a locally regulated intracrine immune pathway. In the endocrine system, cholecalciferol undergoes hepatic conversion to 25(OH)D, which circulates as the major storage form of the hormone [10]. The kidney then converts 25(OH)D to the active metabolite 1,25(OH)_2_D through the vitamin D-activating enzyme CYP27B1, and this renal calcitriol enters the systemic circulation to regulate calcium and phosphate homeostasis in target organs such as bone, intestine, and kidney [10]. In contrast, many immune cells, including dendritic cells, macrophages, and activated T lymphocytes, express CYP27B1 and the vitamin D receptor (VDR), enabling them to convert circulating 25(OH)D into calcitriol locally within the cell [11]. This process constitutes an intracrine signaling pathway in which the active metabolite is synthesized and acts within the same cellular microenvironment rather than being released into the systemic circulation. Unlike renal calcitriol production, which is tightly controlled by parathyroid hormone and calcium–phosphate balance, immune-cell CYP27B1 expression is primarily regulated by inflammatory signals such as Toll-like receptor (TLR) activation and cytokines [11]. As a result, immune cells rely on the availability of circulating 25(OH)D as a substrate for local calcitriol generation, allowing vitamin D signaling to function as a context-dependent modulator of immune responses during inflammation and tissue injury [12]. These biological distinctions explain why circulating 25(OH)D, rather than calcitriol, is the clinically relevant biomarker of vitamin D status. Serum calcitriol concentrations are tightly homeostatically regulated and often remain within the normal range even in the presence of substantial vitamin D deficiency because renal production can transiently compensate by increasing conversion efficiency [13]. Consequently, circulating calcitriol levels provide little information about the availability of substrate for local immune-cell activation of the vitamin D pathway. In contrast, serum 25(OH)D reflects the size of the systemic vitamin D reservoir from which immune cells generate calcitriol through intracrine mechanisms [14]. When circulating 25(OH)D concentrations fall below sufficient levels, immune cells may become substrate-limited, reducing their capacity to generate local calcitriol and to engage VDR-dependent transcriptional programs that regulate antigen presentation, cytokine production, and regulatory T-cell differentiation [14]. The commonly used threshold of approximately 30 ng/mL is derived from endocrine and immunologic studies indicating that concentrations below this range are associated with increased parathyroid hormone activity, impaired skeletal health, and diminished substrate availability for extra-renal vitamin D metabolism [13].

Interpretation of vitamin D thresholds in HSCT populations generally follows endocrine and bone health guidelines. Serum 25(OH)D concentrations <20 ng/mL (50 nmol/L) are widely considered deficient and are associated with impaired calcium absorption, secondary hyperparathyroidism, and reduced bone mineralization, whereas levels of 20–29 ng/mL are typically classified as insufficient. Concentrations >40 ng/mL are generally regarded as safe and may provide additional skeletal or immunologic benefit in some contexts, although consistent clinical advantages above 30 ng/mL remain uncertain. Vitamin D toxicity is uncommon and usually occurs only with sustained levels >150 ng/mL, which can lead to hypercalcemia, hypercalciuria, and renal impairment [15].

Damage-associated molecular patterns including adenosine triphosphate (ATP), high mobility group box-1 protein (HMGB1), and extracellular nucleic acids released during conditioning-induced tissue injury activate innate immune sensors such as TLRs and inflammasome pathways in APCs. Engagement of these pathways induces the production of pro-inflammatory cytokines including tumor necrosis factor-α (TNF-α), interleukin-1β (IL-1β), and interferon-γ (IFN-γ), establishing the cytokine milieu that precedes aGVHD [16]. Importantly, these inflammatory signals also intersect with vitamin D metabolism within immune cells. As previously said, the activation of TLR signaling in human monocytes and macrophages up-regulates expression of both VDR and CYP27B1, thereby enabling the intracrine conversion of circulating 25(OH)D into 1,25(OH)_2_D. This locally generated hormone subsequently drives transcription of vitamin D-responsive genes and promotes regulatory immune programs that limit excessive inflammatory activation [16]. Importantly, this pathway is substrate-dependent: TLR-mediated induction of downstream antimicrobial and immunomodulatory responses occurs efficiently only when sufficient circulating 25(OH)D is available to support CYP27B1-mediated conversion. In this context, vitamin D signaling functions as a counter-regulatory circuit that dampens excessive antigen presentation, reduces co-stimulatory signaling, and favors regulatory cytokine profiles.

**Figure 1:**
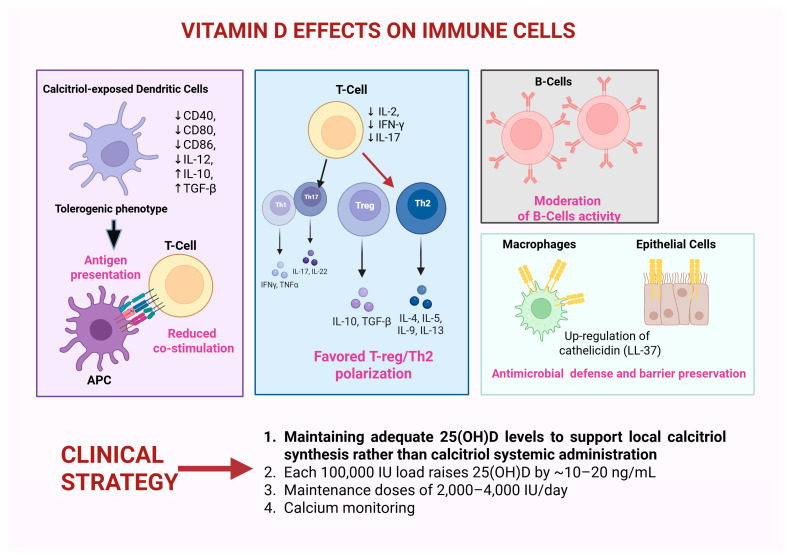
Vitamin D modulates immune reconstitution after hematopoietic stem cell transplantation. Calcitriol promotes tolerogenic dendritic cells, reduces co-stimulation during antigen presentation, and limits alloreactive T-cell responses by down-regulating IL-2, IFN-γ, and IL-17 while favoring Treg/Th2 polarization. B-cell activity is moderated, whereas antimicrobial defense is preserved through cathelicidin (LL-37) induction. Clinical strategies focus on maintaining adequate 25(OH)D to support local calcitriol synthesis rather than systemic administration. Created in Biorender. Manlio Fazio, (2026) https://app.biorender.com/illustrations/696a7c98c004f77154cec9dc.

Calcitriol-exposed monocyte-derived dendritic cells adopt an immature, tolerogenic phenotype with reduced expression of CD40, CD80, and CD86, lower interleukin-12 production, enhanced interleukin-10 and TGF-β, and induction of indoleamine 2,3-dioxygenase [17]. Functionally, antigen presentation proceeds with diminished co-stimulation and reduced alloreactive proliferation in mixed lymphocyte reactions [18]. In T cells, vitamin D receptor signaling down-regulates IL-2, IFN-γ, and IL-17 and tempers Th1/Th17 polarization while favoring regulatory and Th2 programs, thereby stabilizing the immune set-point during reconstitution [19]. B-cell proliferation and immunoglobulin synthesis may also be moderated [20]. Importantly, vitamin D does not act as a global immunosuppressant: macrophages and epithelial cells up-regulate antimicrobial peptides such as cathelicidin (LL-37), and barrier restitution appears indirectly supported [21]. Because immune cells locally synthesize 1,25-dihydroxyvitamin D from the circulating reservoir, clinical programs focus on measuring and correcting 25(OH)D levels rather than routinely prescribing systemic calcitriol to support immune regulation safely [11]. In adults, each 100,000 IU load typically raises 25(OH)D by roughly 10–20 ng/mL, but obesity, systemic inflammation, glucocorticoids, and malabsorption blunt responses [22]. During mucositis or gastrointestinal (GI) GVHD, oral thin-film or intramuscular cholecalciferol can bypass bile-dependent micellar uptake [23]. At maintenance doses of 2000–4000 IU/day, safety is favorable, though calcium monitoring is prudent in renal impairment and granulomatous disease [24,25] (Figure 1).

Beyond dendritic cells and conventional T-cell subsets, vitamin D signaling also shapes the broader cellular network that governs immune synapse formation and microenvironmental stability after allogeneic transplantation. Macrophages represent a critical node in this network, as their polarization states influence both antigen presentation and tissue repair. More specifically, engagement of the VDR in monocytes and macrophages modulates transcriptional programs that favor a regulatory or M2-like phenotype while constraining pro-inflammatory M1 polarization [26]. Mechanistically, calcitriol signaling suppresses NF-κB activation and reduces transcription of inflammatory mediators such as IL-6, TNF-α, and IL-12, while promoting expression of IL-10, arginase-1, and other genes associated with tissue-repair programs [27]. This shift alters the cytokine gradient within the transplant microenvironment, indirectly limiting Th1 and Th17 expansion and attenuating allo-reactive amplification loops. In parallel, vitamin D signaling affects the differentiation and effector behavior of innate lymphoid cells (ILCs), which are increasingly recognized as early regulators of mucosal integrity during GVHD [28]. Data suggest that VDR activation restrains the pro-inflammatory activity of ILC1 and ILC3 subsets by reducing production of interferon-γ and IL-17/IL-22, while preserving regulatory circuits that support epithelial barrier maintenance [29,30]. Because ILCs respond rapidly to alarmins [31] released during conditioning-induced tissue injury (such as IL-33 and IL-25), vitamin D-dependent modulation of their cytokine output may influence the balance between inflammatory amplification and mucosal repair in the earliest stages of GVHD pathogenesis. Through these coordinated effects on macrophage polarization, ILC activity, and downstream T-cell programming, vitamin D acts as a contextual regulator of the immune synapse that integrates innate sensing with adaptive immune calibration in the post-transplant microenvironment.

Genetic variation in the VDR may further influence transplant outcomes. Several polymorphisms, including FokI, BsmI, ApaI, and TaqI, have been associated with differences in immune regulation and susceptibility to GVHD [32]. These variants may alter VDR transcriptional activity and downstream cytokine signaling, potentially modulating T-cell responses and inflammatory pathways relevant to GVHD pathogenesis. For example, observational studies in allo-HSCT cohorts have reported associations between recipient ApaI aa and FokI FF genotypes and an increased risk of aGVHD, while donor ApaI AA and FokI FF genotypes have also been linked to a higher incidence of grades II–IV aGVHD in multivariate analyses. In addition, the recipient ApaI aa genotype has been associated with poorer post-transplant survival, suggesting that enhanced VDR activity may influence immune activation and inflammatory responses during early immune reconstitution [32]. Although findings remain heterogeneous, these observations suggest that both host and donor VDR genotype could contribute to interindividual variability in vitamin D-mediated immune effects after HSCT and potentially influence transplant complications.

## 2. Epidemiology and Clinical Implications of Vitamin D in HSCT

### 2.1. Epidemiology Before and After HSCT

Acute and chronic GVHD represent distinct but overlapping immunopathological syndromes following allogeneic hematopoietic stem cell transplantation [33]. Acute GVHD typically manifests within the first 100 days post-transplant and is driven by donor T-cell recognition of host alloantigens, leading to a cascade of inflammatory cytokine release and tissue damage primarily affecting the skin, gastrointestinal tract, and liver [34]. Chronic GVHD, which may occur de novo or evolve from aGVHD, reflects a more complex interplay of alloimmunity and autoimmunity, characterized by aberrant B-cell activation, fibrosis, and multi-organ involvement resembling autoimmune disorders [35]. Both forms of GVHD remain major determinants of transplant-related morbidity and mortality, despite advances in prophylaxis and immunomodulatory strategies [36].

Across adult cohorts, baseline 25(OH)D concentrations often cluster around 16–20 ng/mL, with 80–90% of patients below 30 ng/mL at admission [37]. Levels frequently decline further by day +30 and day +100, especially in those receiving high-dose corticosteroids for aGVHD or experiencing prolonged gastrointestinal toxicity [38]. Pediatric series echo this pattern, with approximately 70% of children deficient or insufficient at baseline or by day +100 despite empiric supplementation policies [39]. Heterogeneity across studies reflects divergent measurement windows, thresholds (<10, <20, <25, <30 ng/mL), and confounding by indication, whereby sicker patients are both more likely to be supplemented and more likely to remain low due to malabsorption and steroid exposure [40]. These complexities support prespecified measurement, repletion, and monitoring time points rather than reliance on any single observational estimate.

### 2.2. Vitamin D and GVHD: Acute and Chronic

Interpretation of the clinical literature linking vitamin D status with GVHD requires careful consideration of methodological heterogeneity across studies. Most available data derive from observational cohort analyses rather than randomized trials, and important differences exist in study design, population size, timing of vitamin D assessment, and analytical methods used to quantify serum 25(OH)D. Some studies rely on pre-transplant baseline measurements [41,42], whereas others incorporate longitudinal sampling during early immune reconstitution, which may more accurately capture dynamic fluctuations in vitamin D status during the high-risk post-conditioning period [43,44]. In addition, assay methodology varies substantially across cohorts, with some studies using automated immunoassays and others employing liquid chromatography–tandem mass spectrometry (LC–MS/MS), which can introduce systematic differences in measured concentrations [45]. Sample sizes also range widely, from single-center cohorts to multicenter registry analyses, and many studies remain susceptible to confounding factors such as steroid exposure, nutritional status, and seasonal variation. Consequently, the available evidence should be interpreted primarily as observational and hypothesis-generating. Links between baseline 25(OH)D and aGVHD are inconsistent and frequently attenuate after adjustment, with pediatric analyses occasionally yielding paradoxical unadjusted patterns that likely reflect seasonality, prophylaxis platforms, and early supplementation [46]. The timing of aGVHD risk between days 30 and 100 coincides with depressed 25(OH)D caused by mucosal injury, reduced intake, and steroid therapy, generating reverse causation that complicates inference [47,48]. In contrast, a more coherent signal has emerged for cGVHD, with independent adult cohorts reporting higher cGVHD, including extensive disease, and in one analysis increased CMV disease among patients with low pre-HSCT 25(OH)D [49]. Adjusted hazard ratios ranging from roughly two to five suggest that profound deficiency is undesirable, though causality is unproven and disease-free survival (DFS) did not clearly improve with higher levels [50]. Overall survival (OS) tended to be poorer among deficient patients [51]. The stronger association observed between vitamin D status and cGVHD compared with aGVHD may reflect differences in the underlying immunopathology of these syndromes. Acute GVHD is primarily driven by early allo-reactive T-cell activation and inflammatory cytokine release occurring shortly after transplantation, processes that are strongly influenced by conditioning intensity and antigen presentation. In contrast, cGVHD involves more complex and sustained immune dysregulation characterized by impaired immune tolerance, aberrant B-cell activation, regulatory T-cell deficiency, and progressive tissue fibrosis. In addition, the longer time course of cGVHD may increase the cumulative impact of sustained vitamin D insufficiency on immune regulation and tissue repair. Together, these mechanisms may provide a biologically plausible explanation for the more consistent associations reported between vitamin D status and cGVHD outcomes [52].

## 3. Vitamin D and Outcomes After Hematopoietic Stem Cell Transplantation

### 3.1. Survival, Relapses, and Engraftment

Adult multicenter cohorts have not consistently linked baseline vitamin D to aGVHD, relapse, or engraftment. Nonetheless, overall mortality appears higher at very low 25(OH)D thresholds (for example, ≤12 ng/mL), implying that severe deficiency may be important even if intermediate endpoints remain neutral [53]. Among patients transplanted for myeloid malignancies, pre-HSCT deficiency below 20 ng/mL has been associated with higher relapse and worse OS, with validation in an independent cohort [54]. Biologically, vitamin D may shape antileukemic immunity and marrow-niche dynamics, offering a plausible pathway for disease-specific associations [55]. Neutrophil and platelet engraftment appear largely insensitive to baseline status in most series, whereas pediatric data more consistently link adequate day +30 25(OH)D with faster day +100 lymphocyte reconstitution across CD4, B cells, and NK cells [46,56].

### 3.2. Infections and Mucosal Defense

Mechanistically, vitamin D supports epithelial restitution and the induction of antimicrobial peptides; these actions could reduce bacterial translocation and opportunistic infections in damaged mucosa [57,58]. While HSCT-specific infection datasets remain modest, the broader transplant literature often associates lower 25(OH)D with higher late-onset infections after adjustment [59]. Given the high prevalence of deficiency and the low cost of correction, proactive monitoring fits within a comprehensive infection-prevention bundle that also includes vaccination, antimicrobial prophylaxis, oral care, and cytopenia management [60].

### 3.3. Bone Health and Survivorship

Early hip-dominant bone loss is a well-recognized complication after allo-HSCT, driven by multiple converging factors including conditioning-induced hypogonadism, prolonged corticosteroid exposure, reduced mobility, inflammatory cytokine signaling, and inadequate nutritional intake [61]. Prospective studies demonstrate that bone mineral density (BMD) declines rapidly during the first post-transplant year, with losses typically ranging from approximately 3–10% at the femoral neck and total hip and somewhat smaller reductions at the lumbar spine. Several studies (reported in Table 1) evaluating vitamin D and calcium supplementation confirm that although correction of deficiency is necessary to maintain skeletal metabolism, BMD loss may still occur despite supplementation, particularly at cortical sites such as the hip [48,62,63,64,65,66].

**Figure 2:**
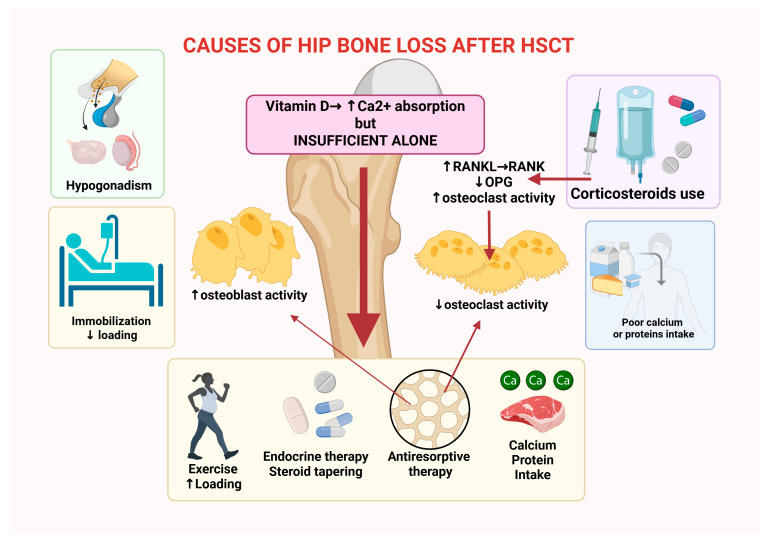
Mechanistic picture of hip bone loss after HSCT. Risk factors such as hypogonadism, corticosteroids, immobilization and poor intake drive: ↓ sex steroids, ↓ loading, ↑ RANKL/osteoclasts, and limited Ca/protein. Vitamin D supports Ca absorption but is insufficient alone. Multimodal care (exercise, endocrine therapy, steroid taper, adequate calcium/protein, antiresorptives) targets these pathways to preserve BMD. Created in Biorender, Manlio Fazio, (2026) https://app.biorender.com/illustrations/696cfbd9970df2a046a25f7f.

In adult randomized cohorts, declines in hip BMD of several percentage points were observed in both intervention and control groups during the first year after transplantation, indicating that vitamin D alone is insufficient to counteract the complex metabolic and treatment-related drivers of post-HSCT bone loss. Long-term observational studies also report an increased risk of fragility fractures among HSCT survivors, particularly in patients with chronic GVHD, prolonged glucocorticoid therapy, or persistent hypogonadism. A survivorship-focused systematic review therefore concluded that evidence supporting fracture reduction with vitamin D and calcium supplementation alone remains of very low certainty, while still recommending routine monitoring and correction of vitamin D deficiency as part of comprehensive survivorship care [47]. Practical bone health management after HSCT is consequently multimodal and includes structured exercise and physiotherapy, endocrine evaluation and treatment of hypogonadism, careful steroid tapering when clinically feasible, adequate calcium intake (1.000–1.200 mg/day) and sufficient protein nutrition, and the use of antiresorptive therapies when indicated [66,67,68] (Figure 2).

## 4. Interventional Strategies and Formulations

### 4.1. Conditioning-Related Pharmacokinetics and Formulation Selection

**Figure 3:**
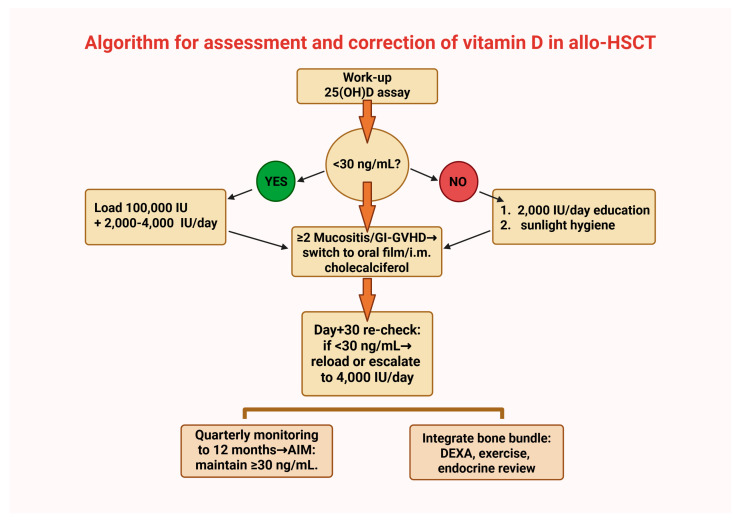
Practical algorithm for assessment and correction of vitamin D in allo-HSCT. Created in Biorender, Manlio Fazio, (2026) https://app.biorender.com/illustrations/696d5dcd7ee8dfc8a17849aa.

A pragmatic strategy for vitamin D repletion in HSCT candidates is to load, reassess, and then maintain. A randomized study comparing 2000 IU/day with or without a single pre-conditioning 100,000 IU loading dose found higher rates of biochemical sufficiency at day +30 with loading, particularly among patients starting from low baselines, although between-arm differences waned by day +100 and the trial was not powered for clinical endpoints [61,69]. A practical pathway measures at work-up, administers loading when <30 ng/mL, initiates 2000–4000 IU/day, reassesses at day +30 with re-loading or escalation if still insufficient, and monitors quarterly through months 3–12 [40,70]. During mucositis or GI GVHD, capsule absorption is unreliable, and oral thin-film or intramuscular cholecalciferol can be used as a bridge until the gut recovers [23,71]. Safety is excellent at these doses, though calcium monitoring is advised in renal impairment and granulomatous disease, and patients should avoid unsupervised mega-dosing [72,73] (Table 2 and Table 3, Figure 3).

Clinical heterogeneity related to conditioning intensity also deserves consideration when interpreting vitamin D pharmacokinetics during the early transplant period. Myeloablative conditioning regimens, particularly those incorporating high-dose alkylating agents or total body irradiation (TBI), produce more profound epithelial injury within the gastrointestinal tract than reduced-intensity or non-myeloablative approaches [34,74]. This injury disrupts tight junction integrity, reduces villous surface area, and impairs bile-mediated micellar solubilization, all of which are required for efficient intestinal absorption of lipophilic molecules such as cholecalciferol [75]. As a consequence, the bioavailability of standard capsule formulations may become highly variable during periods of severe mucositis or early GI GVHD. In such contexts, alternative delivery strategies can offer pharmacokinetic advantages. Oral thin-film preparations dissolve directly on the buccal or sublingual mucosa and allow partial transmucosal absorption that bypasses luminal dissolution and bile-dependent uptake, thereby reducing reliance on an intact intestinal barrier. Intramuscular cholecalciferol represents a complementary strategy when oral intake is unreliable, providing slow release from intramuscular depots and more predictable restoration of circulating 25-hydroxyvitamin D concentrations during periods of gastrointestinal dysfunction [76]. Nevertheless, formulation selection must be balanced against individual metabolic risk. Patients with renal impairment, granulomatous disease, or dysregulated calcium–phosphate homeostasis may have altered conversion of vitamin D metabolites and a greater susceptibility to hypercalcemia following high-dose repletion [11]. In these individuals, cautious dosing with closer biochemical surveillance of calcium, creatinine, and phosphate levels is advisable. Integrating conditioning intensity, gastrointestinal toxicity, and metabolic risk into supplementation algorithms may therefore improve the reliability and safety of vitamin D repletion strategies during the early phases of immune reconstitution after transplantation.

Overall, the strength of evidence supporting vitamin D supplementation in HSCT remains moderate, as most available data derive from observational cohorts and small randomized trials, with few large-scale randomized studies evaluating clinical endpoints; therefore, adequately powered randomized trials are warranted to clarify its impact on transplant outcomes.

### 4.2. Pediatrics and Special Populations

Children undergoing HSCT frequently transition from vitamin D sufficiency before transplantation to insufficiency by approximately day +100 post-transplant [26]. Several physiological factors contribute to this decline. Pediatric patients experience rapid skeletal growth and bone remodeling, which increases vitamin D utilization for calcium absorption and mineralization. In addition, younger children often have lower baseline vitamin D stores, variable nutritional intake, and reduced sunlight exposure during prolonged hospitalization. Post-transplant medications (corticosteroids and calcineurin inhibitors) may further impair vitamin D metabolism and bone turnover [46]. Age-specific pharmacokinetics also influence vitamin D requirements. Because vitamin D is fat-soluble, its distribution and storage differ across developmental stages, with larger relative extracellular fluid volumes and ongoing tissue accretion in growing children. These factors may lead to greater variability in serum 25-hydroxyvitamin D responses to supplementation compared with adults. Growth phases such as early childhood and adolescence, characterized by accelerated skeletal accrual, may therefore require closer biochemical monitoring and dose adjustment. Achieving adequate vitamin D levels by day +30 after HSCT has been associated with faster recovery of CD4^+^ T cells, B cells, and NK cells by day +100, providing a potential immunologic rationale for early correction of deficiency [46]. Practical pediatric dosing combines weight-based daily regimens with supervised stoss loading when adherence is a barrier, always coupled to biochemical monitoring [69,77]. Additional considerations apply to special populations. In children with obesity, higher or repeated loading doses may be required due to sequestration of vitamin D in adipose tissue. In patients with cholestasis or severe mucositis, which may impair oral absorption, alternative formulations such as thin-film preparations or intramuscular administration can be considered [78,79]. Pregnancy after HSCT warrants more conservative targets and multidisciplinary co-management [80].

## 5. Clinically Oriented Operational Guidance

Implementation science principles can accelerate adoption of vitamin D protocols in HSCT without overburdening teams, provided the pathway is framed as a pragmatic, safety-guarded component of routine care [52]. The central clinical aim is straightforward: achieve and maintain serum 25-hydroxyvitamin D [25(OH)D] ≥30 ng/mL by day +30, then sustain trajectories through day +100 and beyond, integrating bone health and immunologic recovery milestones [48]. Reliable attainment of this target hinges on four operational drivers [23]:

1. Timely baseline assays
2. Reflex loading when values are <30 ng/mL
3. Early identification of malabsorption risks with formulation matching
4. Structured re-measurement at prespecified intervals

Mapping each driver to testable change ideas, prechecked order sets in computerized provider order entry (CPOE), pharmacy flags that automatically convert capsules to thin-film in GI-GVHD, and default reminders tied to day +30 labs enables disciplined Plan-Do-Study-Act cycles [81]. Local champions in nursing, pharmacy, and medicine close the loop by owning metrics, coaching peers through early iterations, and maintaining momentum via audit-and-feedback that celebrates high timely sufficiency rates. Clinically, the mechanism supports the operational target without necessitating systemic calcitriol: vitamin D functions as a context-dependent rheostat rather than a binary switch [82]. In inflamed tissue microenvironments, dendritic cells up-regulate CYP27B1 and increase local conversion of 25(OH)D to 1,25(OH)_2_D; this intracrine surge tunes antigen presentation, reduces co-stimulatory density, and enhances IL-10 release [12,83]. At the alloimmune synapse, even modest shifts in co-stimulation and cytokine polarity can influence the balance between effector amplification and regulatory containment [84,85]. Maintaining an adequate circulating reservoir of 25(OH)D allows these circuits to operate within their intended physiologic range, avoiding the hypercalcemia risk that accompanies systemic calcitriol [86].

While vitamin D-mediated signaling represents a physiologic strategy for recalibrating immune tolerance after HSCT, its immunomodulatory profile shares conceptual parallels with several emerging targeted and cellular therapeutic approaches that aim to reshape the transplant microenvironment. Similar to immunomodulatory paradigms described in solid organ transplantation, including liver transplantation [87], vitamin D influences immune synapse dynamics by simultaneously dampening APC activation and promoting regulatory lymphocyte networks. In this context, vitamin D receptor (VDR) signaling in dendritic cells reduces expression of co-stimulatory molecules such as CD80 and CD86 while promoting tolerogenic phenotypes that facilitate expansion of regulatory T cells, mechanisms that mirror tolerance-inducing strategies explored in liver transplant immunotherapy, where modulation of antigen presentation and regulatory cell expansion represents a central objective of immune control [87]. In contrast to pharmacologic kinase inhibitors that directly interrupt intracellular inflammatory cascades, such as suppression of MAPK or ERK phosphorylation pathways involved in neutrophil activation and inflammatory degranulation, vitamin D acts upstream at the transcriptional level, integrating signals that modulate both innate and adaptive immune programs rather than targeting a single signaling node. A complementary strategy is represented by mesenchymal stem cell (MSC)-based therapies, which exert multidimensional regulatory effects within damaged mucosal environments [88]. MSCs derived from umbilical cord or BM have been shown to promote epithelial barrier repair, suppress neutrophil activation, and attenuate local cytokine amplification through mechanisms including inhibition of ERK phosphorylation, secretion of anti-inflammatory mediators such as prostaglandin E2, indoleamine-2,3-dioxygenase and TGF-β, and enhancement of epithelial regeneration [89]. Notably, these reparative mechanisms are particularly relevant in the gastrointestinal tract, a major target organ in GVDH, where disruption of epithelial integrity amplifies immune activation and microbial translocation [88]. Compared with MSC therapy, which primarily provides structural and paracrine support for barrier repair, and kinase-targeting drugs that suppress discrete inflammatory signaling cascades, vitamin D can be viewed as a systemic immunoregulatory cofactor that integrates metabolic sensing with immune cell differentiation. Through coordinated modulation of dendritic cells, macrophage polarization, regulatory T-cell expansion, and epithelial immune responses, vitamin D contributes to the restoration of immune equilibrium within the transplant microenvironment. Examining these complementary strategies across cellular, molecular, and tissue-repair dimensions therefore highlights how distinct immunomodulatory approaches converge on a common objective: re-establishing immune tolerance while preserving protective host defense in complex post-transplant inflammatory settings.

The timing of repletion therefore matters: from conditioning to day +30, patients face barrier disruption, altered bile flow, erratic intake, and fluctuating steroid exposure, precisely when 25(OH)D tends to fall and the immunologic stakes (early tissue injury, innate sensing, antigen presentation, and first-wave alloreactivity) are high [48]. Loading before conditioning produces a step-change in the reservoir that pre-empts the nadir; reassessment at day +30 validates whether maintenance dosing sufficed in the face of real-world malabsorption and catabolism. Programs that wait for spontaneous recovery often find that it lags behind clinical needs [37,46,48]. Bedside decision-making benefits from a simple mental model:

- Think in ng/mL increments
- Match formulation to absorption
- Monitor at prespecified time points

A patient admitted in winter with baseline 25(OH)D 16 ng/mL is unlikely to reach ≥30 ng/mL by day +30 with 2000 IU/day alone; a one-time pre-conditioning load (e.g., 100,000 IU) followed by high-end maintenance (2000–4000 IU/day, selecting the upper range for obesity, winter admissions, or ongoing steroids) and then day +30 biochemistry is safer and more reliable [90,91]. Conversely, a patient with intact intake and baseline 28 ng/mL may reach targets with daily dosing and sunlight hygiene. In GI-GVHD or persistent cholestasis/diarrhea, thin-film oral cholecalciferol, delivered weekly or biweekly and titrated to measured 25(OH)D, bypasses bile-dependent micellar solubilization required by capsules; when oral intake is not feasible, intramuscular dosing serves as a defensible temporary bridge, provided calcium is checked after injections and weekly totals remain conservative [23,70,92]. As gut function normalizes, patients can transition back to capsules at daily maintenance doses. Pharmacokinetic intuition supports these choices: compartment models treat liver and adipose stores as reservoirs with slow release, and a single 100,000 IU dose typically raises serum 25(OH)D by approximately 10–20 ng/mL, with attenuated responses in inflamed states, obesity, and concurrent glucocorticoids [93]. For patients at the lower extreme of baseline values or with persistent malabsorption, repeated loading may be necessary if day +30 measurements show insufficient response; each decision is anchored in measured biochemistry rather than assumed absorption [48]. Assay pragmatics and data integrity underpin interpretation and safe action. Laboratories should confirm assay methods and units in every report, and electronic health records ought to surface both ng/mL and nmol/L with automatic conversions to prevent miscommunication [94]. Immunoassays are accessible but may show small biases compared with liquid chromatography–tandem mass spectrometry (LC-MS/MS), particularly around decision cut-points; transplant centers should strive for within-method consistency across serial measurements and avoid over-interpreting values that hover at thresholds during dynamic clinical periods [95]. When health systems change reference laboratories or platforms, a short bridging exercise, paired sampling across methods, stabilizes longitudinal interpretation and allows teams to adjust thresholds if necessary. Because borderline values can oscillate near decision points, clinicians should prioritize trajectories over isolated measurements and schedule re-checks within 2–4 weeks when values are near 30 ng/mL or formulations have changed [76]. Safety guardrails are simple and effective when codified in order sets. Programs should: (1) document baseline calcium, albumin, and creatinine; (2) re-check calcium after re-loading or intramuscular dosing; (3) discourage unsupervised mega-dosing [76]. Symptoms of hypercalcemia warrant urgent evaluation and temporary cessation [24]. In practice, adverse biochemical events are uncommon at 2000–4000 IU/day, and the risk–benefit calculus strongly favors avoiding severe deficiency while maintaining vigilance [72]. Special populations require nuance [96,97,98,99,100]:

1. Obesity may necessitate larger or repeated loads;
2. Renal impairment and granulomatous disease justify tighter biochemical surveillance after re-loading or intramuscular dosing;
3. Pregnancy after HCT warrants conservative targets and coordinated obstetric–hematology oversight;
4. Pediatrics (where reduced outdoor exposure, altered diet, and steroid pulses can drive declines below 30 ng/mL by day +100) benefit from weight-based daily dosing, supervised stoss regimens when trajectories falter, and child-friendly thin-film formulations that reduce pill-burden friction [96,97,98,99,100].

Operational sustainability and measurement should be put into practice from the start. Program leaders can align incentives by embedding vitamin D sufficiency into quality dashboards alongside neutropenic fever bundles and vaccination timelines. Month-over-month run charts [101] that reveal the percentage of patients at ≥30 ng/mL by day +30, stratified by diagnosis and prophylaxis platform, make variation visible and actionable. When performance dips, annotated charts often reveal proximal causes (seasonal shifts with more winter admissions, staff turnover affecting counseling or temporary thin-film stock constraints), prompting targeted fixes rather than global overhauls. Additional indicators, such as mean change from baseline to day +100, incidence of hypercalcemia, and bone mineral density trajectories at 6–12 months, keep teams aligned with survivorship goals while immune reconstitution proceeds [48]. Education materials merit deliberate design: a two-tier set, one handout for clinicians summarizing thresholds, default actions, and safety checks; another for patients explaining units, timelines, and symptoms of hypercalcemia, reduces noise. Visual timelines that place “baseline” and “day +30” on a single line, with icons for loading, malabsorption switches, and bone bundle initiation, help families situate vitamin D within the broader transplant arc, and multilingual versions minimize disparities [102,103]. Training can be light-touch yet effective: a 20 min onboarding module that orients new staff to the rationale, default orders, and safety guardrails, followed by quarterly refreshers anchored to recent run charts, cultivates shared ownership [102,104]. Pharmacy can maintain par levels for thin-film and intramuscular formulations that anticipate seasonal admission spikes, and formulary policies can tie access to objective triggers (mucositis, GI-GVHD, persistent diarrhea, cholestasis), so scarce resources align with the patients most likely to benefit [23]. Interpreting conflicting observational signals becomes easier once “windowing” and reverse causation are acknowledged: for aGVHD, studies that rely solely on baseline 25(OH)D without serial measurements may miss precipitous declines during the peak hazard window, while steroid therapy for emerging GVHD both depresses 25(OH)D [70,105] and confounds associations. When analyses instead focus on cGVHD, where hazards unfold over months, a coherent pattern emerges: very low pre-HCT levels associate with more extensive disease and, in some series, higher CMV disease [49]. The clinical lesson is modest and actionable: vitamin D is not GVHD prophylaxis, but avoidable deficiency should not coexist with high-risk reconstitution states. Bone health considerations intertwine with the vitamin D story but extend beyond it; early hip-predominant loss reflects hypogonadism, high-dose steroids, immobilization, and suboptimal intake [106]. The remedy is bundled rather than uni-agent: physiotherapy with progressive loading, endocrine evaluation and treatment, targeted nutrition for calcium and protein, and antiresorptives when indicated, embedded into the quarterly monitoring rhythm (months 3–12) [106]. Adherence and communication are pivotal, particularly in pediatrics, where complex regimens in the early outpatient phase can erode adherence to daily capsules; thin-film delivery improves palatability, and stoss regimens can rescue slow trajectories when supervised and coupled to biochemistry [23,69]. Furthermore, framing matters: explaining that day +30 adequacy is linked with faster CD4/B/NK recovery by day +100 helps families perceive vitamin D as part of a coherent plan for immune recovery rather than as an optional supplement.

Finally, implementation is strengthened by case-based protocols that map common trajectories and embed decision points (Table 4).

An adult AML recipient admitted in late-winter with baseline 25(OH)D 18 ng/mL, BMI 32, and prior cholestasis should trigger a one-time pre-conditioning load of 100,000 IU followed by 4000 IU/day, with thin-film as backup if capsules fail; on day +30, 25(OH)D and calcium are rechecked, and if still <30 ng/mL, teams either reload or continue high-end maintenance with close follow-up, moving to thin-film weekly if GI symptoms persist; by day +100, the goal is biochemical stability ≥30 ng/mL and initiation of the bone bundle with DEXA and exercise planning. In steroid-refractory GI-GVHD with persistent diarrhea and cholestasis, staff should assume malabsorption rather than test it repeatedly; thin-film cholecalciferol delivered weekly or biweekly and titrated to measured 25(OH)D reduces variability, and intramuscular dosing is a defensible temporary bridge with post-dose calcium checks and conservative weekly totals; normalizing stools and cholestatic indices permit transition back to oral capsules at daily maintenance. In pediatrics, an ALL recipient with baseline sufficiency may drift below 30 ng/mL by day +100 owing to reduced outdoor exposure, altered diet, and steroid pulses [107,108]; weight-based daily dosing remains the backbone, but supervised stoss regimens can recapture trajectories when coupled to close biochemical monitoring, and explicit goals (“≥30 ng/mL by day +30”) linked to tangible immunologic milestones support adherence [46,69]. Health-economic considerations favor this pragmatic repletion model: the direct costs of measuring 25(OH)D at two prespecified time points and providing loading plus maintenance are modest compared with the potential consequences of persistent deficiency (prolonged steroid exposure for cGVHD, slower immune reconstitution requiring additional clinic visits, and bone loss necessitating therapy) [109,110]. Even absent definitive outcome trials, centers can model savings from avoided phone calls, emergency visits related to cramps or fatigue, and shortened counseling time once standardized materials are deployed, with conservative and transparent assumptions. Ultimately, the goal is not to medicalize a nutrient but to remove avoidable deficiency during a period when the immune system is exquisitely plastic; with calibrated dosing, thoughtful formulation choices, and routine monitoring, transplant centers can operationalize a low-risk, biologically plausible intervention that respects competing clinical priorities and leverages existing workflows rather than creating new ones.

## 6. Future Perspectives

### Research Agenda: Trial Design and Reporting Standards

Research priorities include [111]:

- Outcome-powered trials stratified by baseline deficiency and disease category, with particular attention to myeloid malignancies;
- Formulation trials comparing capsules, oral thin-film, and intramuscular dosing in malabsorption with pharmacokinetic and pharmacodynamic readouts;
- Pediatric precision-dosing studies linking trajectories of 25(OH)D to immune reconstitution and growth;
- Biomarker-embedded cGVHD studies that incorporate serial 25(OH)D and VDR polymorphisms to parse prognostic from predictive roles;
- Factorial “bone bundles” that quantify additive benefits of vitamin D, calcium, exercise, endocrine optimization, and antiresorptives on bone mineral density and fractures [111].

Future event-driven or cluster-pragmatic designs across multiple centers should consider co-primary endpoints such as one-year cGVHD-free survival and a blinded infection composite. Serial 25(OH)D at baseline, day +30, and day +100 should be prespecified, with standardized thresholds and adjustment for adiposity, latitude and season, steroid exposure, and prophylaxis. Authors should report assay methods, adherence, dosing formulations, co-interventions, and safety. Patient-reported outcomes and health-economic analyses will capture benefits beyond traditional endpoints.

Post-transplant cyclophosphamide, abatacept, and calcineurin-sparing platforms have reduced severe aGVHD and are reshaping cGVHD epidemiology [112,113,114]. Vitamin D should be positioned as adjunctive supportive care that may optimize mucosal healing and immune set-points. Trials evaluating vitamin D algorithms must be nested within modern prophylaxis and prespecify how these platforms modify endpoints and mechanistic readouts.

Finally, accumulating evidence suggests that sex- and gender-related factors may modulate both vitamin D biology and post-transplant outcomes. Sex differences in vitamin D synthesis, metabolism, and VDR signaling have been described, with women in some settings showing higher 1,25(OH)_2_D levels and potentially greater VDR responsiveness, whereas men may display lower circulating metabolites under similar exposures; these patterns could translate into differential immune modulation and tissue repair after HSCT [115]. In a recent HSCT cohort, peri-transplant 1,25(OH)_2_D concentrations were higher in female recipients, yet the association between active metabolite levels and one-year transplant-related mortality held in both sexes, underscoring the biological plausibility of sex-specific strata without excluding shared risk mechanisms [116]. Beyond transplantation, reviews highlight sex-dependent crosstalk between vitamin D and sex hormones, with estrogens up-regulating VDR expression and enhancing anti-inflammatory effects, suggesting that deficiency (highly prevalent after HSCT) might differentially impact outcomes in women and men and warrant sex-stratified targets and analyses in future trials [117,118,119].

However, observational studies dominate the literature and are vulnerable to residual confounding and reverse causation. Assay variability between immunoassay and LC–MS/MS can misclassify patients around practical thresholds, and heterogeneity in case mix, latitude, and prophylaxis complicates synthesis.

The practical guidance offered here (screen, replete and monitor) rests on safety and biological plausibility rather than proven outcome benefit and should be embedded within comprehensive supportive care.

## 7. Conclusions

Vitamin D deficiency is ubiquitous in allo-HSCT and intersects mechanistically with antigen presentation, T-cell programming, antimicrobial defense, and bone turnover. The most reproducible clinical signal links low pre-HSCT 25(OH)D with cGVHD and, in myeloid diseases, higher relapse; pediatric data associate severe day +100 deficiency with inferior survival and inadequate day +30 levels with slower lymphocyte recovery. Loading before conditioning improves early biochemical sufficiency, especially from low baselines, but outcome benefits remain unproven. Until definitive trials report, targeting ≥30 ng/mL by day +30 with loading-plus-maintenance dosing, switching to absorption-savvy formulations when the gut is compromised, and embedding vitamin D within bone and survivorship bundles constitute reasonable, low-risk steps.

## Figures and Tables

**Table 1:** Summary of BMD changes and skeletal outcomes reported in clinical studies of HSCT.

Study	Study Design/ Population	Sample Size	Follow up	BMD Change (%)	Skeletal Outcomes
Saunders et al. (2023) [65]	Review summarizing observational HSCT cohorts	Multiple cohorts	6–120 months	Femoral neck: 6.2–12% loss first year; Lumbar spine: 3–17% loss; Whole body: ~3.1–3.8% loss	Osteopenia prevalence 29–75% (spine); 33–59% (femoral neck)
Miglietta et al. 2023) [66]	Prospective randomized study evaluating vitamin D and calcium supplementation after HSCT	~150 adult patients	12 months	Continued hip BMD decline despite supplementation (several percentage points)	No significant fracture prevention demonstrated
Pundole et al. (2019) [64]	Meta-analysis of bisphosphonate therapy after HSCT	643 patients	12 months	Lumbar spine MD +7.8% vs. control; Femoral neck MD +6.7% vs. control	Reduced bone loss with anti-resorptive therapy
Saunders et al. 2023) [65]	Observational studies of multiple cohorts of HSCT survivors	Multiple HSCT cohorts	Up to 5 years	Progressive femoral neck decline may persist for 48–120 months after HSCT	Avascular necrosis 4–19%; cumulative incidence 3–10% at 5 years
Panditi et al. (2015) [63]	Prospective cohort	25 HSCT recipients (19 allogeneic, 6 autologous)	Baseline, 3–6 months, 12 months post-HSCT	At 6 months: −8.7% total femur, −5.0% femoral neck, −6.0% trochanter, −9.9% Ward’s triangle; smaller decline at lumbar spine (−2.7%, NS). Partial recovery by 12 months except at Ward’s triangle	Fractures not reported; biochemical markers showed ↓ osteocalcin and ↑ N-telopeptide at 3–6 months, normalizing by 12 months
Schulte et al. (2004) [62]	Prospective single-center cohort of patients undergoing allogeneic HSCT, evaluating long-term changes in BMD with vitamin D and calcium supplementation	280 patients	Baseline and annually for ≥4 years	Average annual loss: −0.6% spine, −0.4% total body, −2.3% femoral neck, −3.5% Ward’s triangle; nadir BMD at 6 months (spine) and 24 months (femoral neck/total body)	Fracture incidence not systematically reported; steroid exposure strongly associated with accelerated bone loss
Bai et al. (2023) [48]	Randomized controlled trial in allogeneic HSCT recipients comparing a vitamin D loading dose (100,000 IU) plus 2000 IU/day vs. 2000 IU/day alone	74 patients	Baseline, day 30, and day 100 post-HSCT	Total hip BMD: −1.7% (loading-dose group) vs. −2.1% (control); Femoral neck: −0.7% (loading-dose group) vs. −2.3% (control)	Fracture outcomes not reported

**Table 2:** Vitamin D status definitions and operational targets in HSCT.

Category	Serum 25(OH)D (ng/mL)	Clinical Note
Severe deficiency	<10	Avoid at work-up/day +30; load + absorption-savvy delivery
Deficiency	<20	Linked to cGVHD; relapse signal in myeloid malignancies
Insufficiency	20–29	Pre-conditioning loading accelerates day +30 sufficiency
Operational target	≥30 by day +30	Pragmatic threshold during immune reconstitution

**Table 3:** Practical dosing pathway for vitamin D in allo-HSCT.

Step	Action
Work-up	Measure 25(OH)D, calcium, albumin, creatinine; if <30 ng/mL → load 100,000 IU + start 2000–4000 IU/day
Day +30	Re-measure 25(OH)D and calcium; if still <30 ng/mL → reload or escalate to 4000 IU/day
Malabsorption	If mucositis ≥ grade 2 or GI-GVHD → switch to oral thin - film weekly or intramuscular cholecalciferol
Months 3–12	Quarterly monitoring; maintain ≥30 ng/mL; integrate bone bundle (DEXA, exercise, endocrine review)
Safety	Avoid unsupervised mega-dosing; check calcium after re-loading/IM dosing; caution in renal impairment/granulomatous disease

Abbreviations: 25(OH)D (25-hydroxycholecalciferol); IU (International Units); GI-GVHD (gastrointestinal graft-versus-host disease); DEXA (Dual-Energy X-ray Absorptiometry).

**Table 4:** Case-based protocols illustrating possible Vitamin D trajectories after HSCT.

Representative Case	Presenting Clinical Profile	Default Protocol Action	Day +30 Decision Point	Subsequent Adjustment
**Adult AML, late-winter admission**	-Baseline 25(OH)D 18 ng/mL; -BMI 32; -Prior cholestasis	One-time pre-conditioning load (e.g., 100,000 IU) + high-end maintenance (4000 IU/day)	Re-check 25(OH)D and Ca^2+^	If <30 ng/mL: repeat load or continue high-end maintenance; Switch to thin-film if GI symptoms persist
**Steroid-refractory** **GI-GVHD**	Persistent diarrhea and cholestasis: unreliable oral absorption	Initiate thin-film cholecalciferol weekly or biweekly, titrated to measured 25(OH)D	Assess trajectory rather than single value; Check calcium after injections	Use intramuscular dosing as temporary bridge if oral intake fails; Transition back to capsules as stools and cholestasis improve
**Pediatric ALL with early sufficiency**	Baseline sufficiency but: - declining intake - reduced sun exposure - steroid pulses	Weight-based daily maintenance dosing	Day +100 25(OH)D reassessment	If <30 ng/mL: supervised stoss regimen with close biochemical follow-up; reinforce adherence with caregivers
**Adult with intact intake and borderline baseline**	Baseline 25(OH)D ~28 ng/mL; no malabsorption	-Daily maintenance dosing (2000 IU/day) -Sunlight hygiene	Day +30 25(OH)D	Continue maintenance if ≥30 ng/mL; escalate dose only if trajectory is insufficient

Abbreviations: AML (acute myeloid leukemia); BMI (body mass index); Ca^2+^ (calcium); GI (gastrointestinal); GVHD (graft-versus-host disease); ALL (acute lymphoblastic leukemia).

## Data Availability

No new data were created or analyzed in this study. Data sharing is not applicable to this article.

## Abbreviations

The following abbreviations are used in this manuscript:

HSCT	Hematopoietic Stem Cell Transplantation
aGVHD	Acute Graft-versus-Host Disease
cGVHD	Chronic Graft-versus-Host Disease
GVHD	Graft-versus-Host Disease
APC	Antigen-Presenting Cell
Treg	Regulatory T cells
Th1	T helper 1 cells
Th2	T helper 2 cells
Th17	T helper 17 cells
NK	Natural Killer cells
CMV	Cytomegalovirus
HLA	Human Leukocyte Antigen
25(OH)D	25-Hydroxyvitamin D
1,25(OH)_2_D	1,25-Dihydroxyvitamin D
VDR	Vitamin D Receptor
CYP27B1	1-alpha-hydroxylase
DEXA	Dual-Energy X-ray Absorptiometry
BMI	Body Mass Index
AML	Acute Myeloid Leukemia
ALL	Acute Lymphoblastic Leukemia
IU	International Units
GI-GVHD	Gastrointestinal Graft-versus-Host Disease
LC-MS/MS	Liquid Chromatography–Tandem Mass Spectrometry
CPOE	Computerized Provider Order Entry
LL-37	Cathelicidin Antimicrobial Peptide
OS	Overall Survival
DFS	Disease-Free Survival

## References

[1] Munshi L., Dumas G., Ferreryro B., Gutierrez C., Böll B., Castro P., Chawla S., Di Nardo M., Lafarge A., McEvoy C. (2025). Contemporary review of critical illness following allogeneic hematopoietic stem cell transplant in adults. Intensive Care Med..

[2] Brennan T.V., Rendell V.R., Yang Y. (2015). Innate Immune Activation by Tissue Injury and Cell Death in the Setting of Hematopoietic Stem Cell Transplantation. Front. Immunol..

[3] Hill G.R., Koyama M. (2020). Cytokines and costimulation in acute graft-versus-host disease. Blood.

[4] Chun R.F., Liu P.T., Modlin R.L., Adams J.S., Hewison M. (2014). Impact of vitamin D on immune function: Lessons learned from genome-wide analysis. Front. Physiol..

[5] Adams J.S., Hewison M. (2010). Update in Vitamin D. J. Clin. Endocrinol. Metab..

[6] Dustin M.L. (2014). The Immunological Synapse. Cancer Immunol. Res..

[7] Bechard L.J., Gordon C., Feldman H.A., Venick R., Gura K., Guinan E.C., Duggan C. (2015). Bone loss and vitamin D deficiency in children undergoing hematopoietic cell transplantation. Pediatr. Blood Cancer.

[8] Flamann C., Peter K., Kreutz M., Bruns H. (2019). Regulation of the Immune Balance During Allogeneic Hematopoietic Stem Cell Transplantation by Vitamin D. Front. Immunol..

[9] Rebelos E., Tentolouris N., Jude E. (2023). The Role of Vitamin D in Health and Disease: A Narrative Review on the Mechanisms Linking Vitamin D with Disease and the Effects of Supplementation. Drugs.

[10] Fraser D.R. (2025). Perspective: Vitamin D Deficiency Relationship to Initiation of Diseases. Nutrients.

[11] Adams J.S., Hewison M. (2012). Extrarenal expression of the 25-hydroxyvitamin D-1-hydroxylase. Arch. Biochem. Biophys..

[12] Bishop E.L., Ismailova A., Dimeloe S.K., Hewison M., White J.H. (2020). Vitamin D and Immune Regulation: Antibacterial, Antiviral, Anti-Inflammatory. J. Bone Miner. Res. Plus.

[13] Giustina A., Bilezikian J.P., A Adler R., Banfi G., Bikle D.D., Binkley N.C., Bollerslev J., Bouillon R., Brandi M.L., Casanueva F.F. (2024). Consensus Statement on Vitamin D Status Assessment and Supplementation: Whys, Whens, and Hows. Endocr. Rev..

[14] Artusa P., White J.H. (2025). Vitamin D and its analogs in immune system regulation. Pharmacol. Rev..

[15] Demay M.B., Pittas A.G., Bikle D.D., Diab D.L., E Kiely M., Lazaretti-Castro M., Lips P., Mitchell D.M., Murad M.H., Powers S. (2024). Vitamin D for the Prevention of Disease: An Endocrine Society Clinical Practice Guideline. J. Clin. Endocrinol. Metab..

[16] Liu P.T., Stenger S., Li H., Wenzel L., Tan B.H., Krutzik S.R., Ochoa M.T., Schauber J., Wu K., Meinken C. (2006). Toll-Like Receptor Triggering of a Vitamin D-Mediated Human Antimicrobial Response. Science.

[17] Penna G., Adorini L. (2000). 1α,25-Dihydroxyvitamin D3 Inhibits Differentiation, Maturation, Activation, and Survival of Dendritic Cells Leading to Impaired Alloreactive T Cell Activation. J. Immunol..

[18] Széles L., Keresztes G., Töröcsik D., Balajthy Z., Krenács L., Póliska S., Steinmeyer A., Zuegel U., Pruenster M., Rot A. (2009). 1,25-Dihydroxyvitamin D3 Is an Autonomous Regulator of the Transcriptional Changes Leading to a Tolerogenic Dendritic Cell Phenotype. J. Immunol..

[19] Harrison S.R., Li D., Jeffery L.E., Raza K., Hewison M. (2020). Vitamin D, Autoimmune Disease and Rheumatoid Arthritis. Calcif. Tissue Int..

[20] Chen S., Sims G.P., Chen X.X., Gu Y.Y., Chen S., E Lipsky P. (2007). Modulatory Effects of 1,25-Dihydroxyvitamin D3 on Human B Cell Differentiation. J. Immunol..

[21] Gombart A.F., Borregaard N., Koeffler H.P. (2005). Human cathelicidin antimicrobial peptide (CAMP) gene is a direct target of the vitamin D receptor and is strongly up-regulated in myeloid cells by 1,25-dihydroxyvitamin D_3_. FASEB J..

[22] Ilahi M., Armas L.A., Heaney R.P. (2008). Pharmacokinetics of a single, large dose of cholecalciferol. Am. J. Clin. Nutr..

[23] Bartlett A.L., Zhang G., Wallace G., McLean S., Myers K.C., Teusink-Cross A., Taggart C., Patel B., Davidson R., Davies S.M. (2023). Optimized vitamin D repletion with oral thin film cholecalciferol in patients undergoing stem cell transplant. Blood Adv..

[24] Billington E.O., Burt L.A., Rose M.S., Davison E.M., Gaudet S., Kan M., Boyd S.K., Hanley D.A. (2020). Safety of High-Dose Vitamin D Supplementation: Secondary Analysis of a Randomized Controlled Trial. J. Clin. Endocrinol. Metab..

[25] Rizzoli R. (2021). Vitamin D supplementation: Upper limit for safety revisited?. Aging Clin. Exp. Res..

[26] Zhang X., Zhou M., Guo Y., Song Z., Liu B. (2015). 1,25-Dihydroxyvitamin D_3_Promotes High Glucose-Induced M1 Macrophage Switching to M2 via the VDR-PPARγSignaling Pathway. BioMed Res. Int..

[27] Aranow C. (2011). Vitamin D and the Immune System. J. Investig. Med..

[28] Sassi F., Tamone C., D’Amelio P. (2018). Vitamin D: Nutrient, Hormone, and Immunomodulator. Nutrients.

[29] He L., Zhou M., Li Y.C. (2019). Vitamin D/Vitamin D Receptor Signaling Is Required for Normal Development and Function of Group 3 Innate Lymphoid Cells in the Gut. iScience.

[30] Konya V., Czarnewski P., Forkel M., Rao A., Kokkinou E., Villablanca E.J., Almer S., Lindforss U., Friberg D., Höög C. (2018). Vitamin D downregulates the IL-23 receptor pathway in human mucosal group 3 innate lymphoid cells. J. Allergy Clin. Immunol..

[31] Ignacio A., Breda C.N.S., Camara N.O.S. (2017). Innate lymphoid cells in tissue homeostasis and diseases. World J. Hepatol..

[32] Bogunia-Kubik K., Middleton P., Norden J., Dickinson A., Lange A. (2008). Association of vitamin D receptor polymorphisms with the outcome of allogeneic haematopoietic stem cell transplantation. Int. J. Immunogenet..

[33] NASSEREDDiNE S., Rafei H., Elbahesh E., Tabbara I. (2017). Acute Graft Versus Host Disease: A Comprehensive Review. Anticancer Res..

[34] Ferrara J.L., E Levine J., Reddy P., Holler E. (2009). Graft-versus-host disease. Lancet.

[35] Zeiser R., Blazar B.R. (2017). Pathophysiology of Chronic Graft-versus-Host Disease and Therapeutic Targets. N. Engl. J. Med..

[36] Pidala J.A., Gooley T.A., Luznik L., Blazar B.R. (2024). Chronic graft-versus-host disease: Unresolved complication or ancient history?. Blood.

[37] Toemthong C., Chanswangphuwana C., Polprasert C., Kongkiatkamon S., Lawasut P., Bunworasate U., Wudhikarn K. (2023). High Incidence of Vitamin D Deficiency Among Patients Undergoing Hematopoietic Stem Cell Transplant and a Single Ultra-High Dose Vitamin D Replacement. Blood.

[38] Sproat L., Bolwell B., Rybicki L., Dean R., Sobecks R., Pohlman B., Andresen S., Sweetenham J., Copelan E., Kalaycio M. (2011). Vitamin D Level after Allogeneic Hematopoietic Stem Cell Transplant. Biol. Blood Marrow Transplant..

[39] Duncan C.N., Vrooman L., Apfelbaum E.M., Whitley K., Bechard L., Lehmann L.E. (2011). 25-Hydroxy Vitamin D Deficiency Following Pediatric Hematopoietic Stem Cell Transplant. Biol. Blood Marrow Transplant..

[40] Wallace G., Jodele S., Myers K.C., Dandoy C.E., El-Bietar J., Nelson A., Taggart C.B., Daniels P., Lane A., Howell J. (2016). Vitamin D Deficiency in Pediatric Hematopoietic Stem Cell Transplantation Patients Despite Both Standard and Aggressive Supplementation. Biol. Blood Marrow Transplant..

[41] Glotzbecker B., Ho V.T., Aldridge J., Kim H.T., Horowitz G., Ritz J., Soiffer R., Avigan D., Rosenblatt J. (2012). Low levels of 25-hydroxyvitamin D before allogeneic hematopoietic SCT correlate with the development of chronic GVHD. Bone Marrow Transplant..

[42] Zahedi H., Khosroshahi R.A., Sadeghi O., Mehdizadeh M., Parkhideh S., Hadizadeh M., Naeini F., Hajifathali A., Shadnoush M. (2024). Association Between Serum Levels of Vitamin D and Biochemical Markers Among Hematopoietic Stem Cell Transplantation Candidates: A Cross-Sectional Study. Int. J. Cancer Manag..

[43] Kamel A.M., Radwan E.R., Zeidan A., Zaky A., Ibrahim A., Refaat A., Abdelfattah R., Abdelfattah M. (2023). Variability of contribution of 1,25 (OH)2D3 (vitamin D) level to hematopoietic stem cell transplantation outcome. Clin. Nutr. ESPEN.

[44] Bhandari R., Malvar J., Sacapano A., Aguayo-Hiraldo P., Jodele S., Orgel E. (2020). Association between Vitamin D and Risk for Early and Late Post-Transplant Complications. Biol. Blood Marrow Transplant..

[45] Wise S.A., Cavalier É., Lukas P., Peeters S., Le Goff C., Briggs L.E., Williams E.L., Mineva E., Pfeiffer C.M., Vesper H. (2025). Commutability assessment of new standard reference materials (SRMs) for determining serum total 25-hydroxyvitamin D using ligand binding and liquid chromatography–tandem mass spectrometry (LC–MS/MS) assays. Anal. Bioanal. Chem..

[46] Bodea J., Beebe K., Campbell C., Salzberg D., Schwalbach C., Miller H., Adams R., Mirea L., Castillo P., Horn B. (2022). Impact of Adequate Day 30 Post-Pediatric Hematopoietic Stem Cell Transplantation Vitamin D Level on Clinical Outcome: An Observational Cohort Study. Biol. Blood Marrow Transplant..

[47] Johnson C., McCormack D., Brown T., MacLaughlin H.L., Blake C., Willims R., Andersen S. (2025). Micronutrient requirements for stem cell transplantation patients > 100 days after transplant and during graft versus host disease: A systematic review. Support. Care Cancer.

[48] Bai N., Lee K., Limvorapitak W., Liu E., Kendler D., Broady R., White J. (2023). Loading dose vitamin D3 improves vitamin D insufficiency in adults undergoing hematopoietic stem cell transplantation: A randomized controlled trial. PLoS ONE.

[49] von Bahr L., Blennow O., Alm J., Björklund A., Malmberg K.-J., Mougiakakos D., Le Blanc A., Oefner P.J., Labopin M., Ljungman P. (2015). Increased incidence of chronic GvHD and CMV disease in patients with vitamin D deficiency before allogeneic stem cell transplantation. Bone Marrow Transplant..

[50] Ito Y., Honda A., Kurokawa M. (2022). Impact of vitamin D level at diagnosis and transplantation on the prognosis of hematological malignancy: A meta-analysis. Blood Adv..

[51] Wallace G., Jodele S., Howell J., Myers K.C., Teusink A., Zhao X., Setchell K., Holtzapfel C., Lane A., Taggart C. (2015). Vitamin D Deficiency and Survival in Children after Hematopoietic Stem Cell Transplant. Biol. Blood Marrow Transplant..

[52] Mancin S., Cangelosi G., Matteucci S., Palomares S.M., Parozzi M., Sandri E., Sguanci M., Piredda M. (2024). The Role of Vitamin D in Hematopoietic Stem Cell Transplantation: Implications for Graft-versus-Host Disease—A Narrative Review. Nutrients.

[53] Yigenoglu T.N., Ulu B.U., Namdaroglu S., Erkurt M.A., Sahin R., Okumus N., Yilmaz S., Ceran F., Koca M., Hatipoglu U. (2024). Is there a relationship between vitamin D levels and graft versus host disease?. Transfus. Apher. Sci..

[54] Radujkovic A., Kordelas L., Krzykalla J., Beelen D.W., Benner A., Lehners N., Schmidt K., Dreger P., Luft T. (2017). Pretransplant Vitamin D Deficiency Is Associated With Higher Relapse Rates in Patients Allografted for Myeloid Malignancies. J. Clin. Oncol..

[55] Kulling P.M., Olson K.C., Olson T.L., Feith D.J., Loughran T.P. (2016). Vitamin D in hematological disorders and malignancies. Eur. J. Haematol..

[56] Raoufinejad K., Shamshiri A.R., Pezeshki S., Chahardouli B., Hadjibabaie M., Jahangard-Rafsanjani Z., Gholami K., Rajabi M., Vaezi M. (2019). Oral calcitriol in hematopoietic recovery and survival after autologous stem cell transplantation: A randomized clinical trial. DARU J. Pharm. Sci..

[57] White J.H. (2022). Emerging Roles of Vitamin D-Induced Antimicrobial Peptides in Antiviral Innate Immunity. Nutrients.

[58] Assa A., Vong L., Pinnell L.J., Avitzur N., Johnson-Henry K.C., Sherman P.M. (2014). Vitamin D Deficiency Promotes Epithelial Barrier Dysfunction and Intestinal Inflammation. J. Infect. Dis..

[59] Fernández-Ruiz M., Corbella L., Morales-Cartagena A., González E., Polanco N., Ruiz-Merlo T., Parra P., Silva J.T., López-Medrano F., Juan R.S. (2018). Vitamin D deficiency and infection risk in kidney transplant recipients: A single-center cohort study. Transpl. Infect. Dis..

[60] Ki M.S., Kim N.E., Woo A., Kim S.Y., Kim Y.S., Kim H.E., Lee J.G., Paik H.C., Park M.S. (2024). Post-Transplant Vitamin D Deficiency in Lung Transplant Recipients: Impact on Outcomes and Prognosis. Transpl. Int..

[61] Kendler D.L., Body J.J., Brandi M.L., Broady R., Cannata-Andia J., Cannata-Ortiz M.J., El Maghraoui A., Guglielmi G., Hadji P., Pierroz D.D. (2018). Bone management in hematologic stem cell transplant recipients. Osteoporos. Int..

[62] Schulte C.M.S., Beelen D.W. (2004). Bone loss following hematopoietic stem cell transplantation: A long-term follow-up. Blood.

[63] Garg M., Pandit A., Kotwal N., Brar K., Gundgurthi A., Sharma A., Sharma S. (2015). Changes in bone mineral density and bone turnover markers in patients undergoing hematopoietic stem cell transplant. Indian J. Endocrinol. Metab..

[64] Pundole X.N., Barbo A.G., Lin H., Champlin R.E., Lu H. (2015). Increased Incidence of Fractures in Recipients of Hematopoietic Stem-Cell Transplantation. J. Clin. Oncol..

[65] Saunders I.M., Tan M., Koura D., Young R. (2020). Long-term Follow-up of Hematopoietic Stem Cell Transplant Survivors: A Focus on Screening, Monitoring, and Therapeutics. Pharmacother. J. Hum. Pharmacol. Drug Ther..

[66] Miglietta F., Iamartino L., Palmini G., Giusti F., Marini F., Iantomasi T., Brandi M.L. (2023). Endocrine sequelae of hematopoietic stem cell transplantation: Effects on mineral homeostasis and bone metabolism. Front. Endocrinol..

[67] Martín-Sánchez C., Polo-Ferrero L., Baile-González M., Marcos-Asensio S., Fernández-Rodríguez E.J., Méndez-Sánchez R., Navarro-López V., Puente-González A.S., López-Corral L., Navarro-Bailón A. (2025). Effects of physical exercise in patients undergoing haematopoietic stem cell transplantation: Systematic review and meta-analysis. Support. Care Cancer.

[68] Kananen K., Volin L., Laitinen K., Alfthan H., Ruutu T., Vaälimaäki M.J. (2005). Prevention of Bone Loss after Allogeneic Stem Cell Transplantation by Calcium, Vitamin D, and Sex Hormone Replacement with or without Pamidronate. J. Clin. Endocrinol. Metab..

[69] Bodea J., Beebe K., Campbell C., Salzberg D., Miller H., Adams R., Mirea L., Castillo P., Horn B., Bansal S. (2021). Stoss therapy is safe for treatment of vitamin D deficiency in pediatric patients undergoing HSCT. Bone Marrow Transplant..

[70] Hong S., Ferraro C.S., Hamilton B.K., Majhail N.S. (2020). To D or not to D: Vitamin D in hematopoietic cell transplantation. Bone Marrow Transplant..

[71] Wylon K., Drozdenko G., Krannich A., Heine G., Dölle S., Worm M. (2017). Pharmacokinetic Evaluation of a Single Intramuscular High Dose versus an Oral Long-Term Supplementation of Cholecalciferol. PLoS ONE.

[72] Zittermann A., Trummer C., Theiler-Schwetz V., Pilz S. (2023). Long-term supplementation with 3200 to 4000 IU of vitamin D daily and adverse events: A systematic review and meta-analysis of randomized controlled trials. Eur. J. Nutr..

[73] Jørgensen H.S., Vervloet M., Cavalier E., Bacchetta J., de Borst M.H., Bover J., Cozzolino M., Ferreira A.C., Hansen D., Herrmann M. (2025). The role of nutritional vitamin D in chronic kidney disease–mineral and bone disorder in children and adults with chronic kidney disease, on dialysis, and after kidney transplantation—A European consensus statement. Nephrol. Dial. Transplant..

[74] Blazar B.R., Murphy W.J., Abedi M. (2012). Advances in graft-versus-host disease biology and therapy. Nat. Rev. Immunol..

[75] Bikle D.D. (2014). Vitamin D Metabolism, Mechanism of Action, and Clinical Applications. Chem. Biol..

[76] Holick M.F., Binkley N.C., Bischoff-Ferrari H.A., Gordon C.M., Hanley D.A., Heaney R.P., Murad M.H., Weaver C.M. (2011). Evaluation, Treatment, and Prevention of Vitamin D Deficiency: An Endocrine Society Clinical Practice Guideline. Med. J. Clin. Endocrinol. Metab..

[77] Bhandari R., Aguayo-Hiraldo P., Malvar J., Cheng K., Sacapano A., Abdel-Azim H., Chi Y.-Y., Wallace G., Asgharzadeh S., Jodele S. (2021). Ultra-High Dose Vitamin D in Pediatric Hematopoietic Stem Cell Transplantation: A Nonrandomized Controlled Trial. Biol. Blood Marrow Transplant..

[78] Tobias D.K., Luttmann-Gibson H., Mora S., Danik J., Bubes V., Copeland T., LeBoff M.S., Cook N.R., Lee I.-M., Buring J.E. (2023). Association of Body Weight With Response to Vitamin D Supplementation and Metabolism. JAMA Netw. Open.

[79] Chongthavornvasana S., Lertudomphonwanit C., Mahachoklertwattana P., Korwutthikulrangsri M. (2023). Determination of Optimal Vitamin D Dosage in Children with Cholestasis. BMC Pediatr..

[80] Zerofsky M.S., Jacoby B.N., Pedersen T.L., Stephensen C.B. (2016). Daily Cholecalciferol Supplementation during Pregnancy Alters Markers of Regulatory Immunity, Inflammation, and Clinical Outcomes in a Randomized Controlled Trial. J. Nutr..

[81] Kenny S.A., Collum K., Featherstone C.A., Farooki A., Jakubowski A. (2019). Impact of a Replacement Algorithm for Vitamin D Deficiency in Adult Hematopoietic Stem Cell Transplant Patients. J. Adv. Pract. Oncol..

[82] Adams J.S., Ren S., Liu P.T., Chun R.F., Lagishetty V., Gombart A.F., Borregaard N., Modlin R.L., Hewison M. (2009). Vitamin D-Directed Rheostatic Regulation of Monocyte Antibacterial Responses. J. Immunol..

[83] Fritsche J., Mondal K., Ehrnsperger A., Andreesen R., Kreutz M. (2003). Regulation of 25-hydroxyvitamin D3-1α-hydroxylase and production of 1α,25-dihydroxyvitamin D3 by human dendritic cells. Blood.

[84] Nishiyama N., Ruoff P., Jimenez J.C., Miwakeichi F., Nishiyama Y., Yata T. (2023). Modeling the interaction between donor-derived regulatory T cells and effector T cells early after allogeneic hematopoietic stem cell transplantation. Biosystems.

[85] Duneton C., Winterberg P.D., Ford M.L. (2022). Activation and regulation of alloreactive T cell immunity in solid organ transplantation. Nat. Rev. Nephrol..

[86] Pike J.W., Meyer M.B. (2020). The unsettled science of nonrenal calcitriol production and its clinical relevance. J. Clin. Investig..

[87] Sensi B., Angelico R., Toti L., Conte L.E., Coppola A., Tisone G., Manzia T.M. (2024). Mechanism, Potential, and Concerns of Immunotherapy for Hepatocellular Carcinoma and Liver Transplantation. Curr. Mol. Pharmacol..

[88] Wang R., Xiong J., Xu Q., Zhou Y., Yang S., Song Q., Wang X., Zhang X. (2025). Mesenchymal Stromal Cells and Graft-versus-Host Disease: Preclinical and Clinical Studies. Stem Cell Rev. Rep..

[89] Wang G., Joel M.D.M., Yuan J., Wang J., Cai X., Ocansey D.K.W., Yan Y., Qian H., Zhang X., Xu W. (2020). Human umbilical cord mesenchymal stem cells alleviate inflammatory bowel disease by inhibiting ERK phosphorylation in neutrophils. Inflammopharmacology.

[90] Kearns M.D., Binongo J.N.G., Watson D., A Alvarez J., Lodin D., Ziegler T.R., Tangpricha V. (2014). The effect of a single, large bolus of vitamin D in healthy adults over the winter and following year: A randomized, double-blind, placebo-controlled trial. Eur. J. Clin. Nutr..

[91] Tóth B.E., Takács I., Kádár K., Mirani S., Vecsernyés M., Lakatos P. (2024). Safety and Efficacy of Loading Doses of Vitamin D: Recommendations for Effective Repletion. Pharmaceuticals.

[92] Kearns M.D., Alvarez J.A., Tangpricha V. (2014). Large, Single-Dose, Oral Vitamin D Supplementation in Adult Populations: A Systematic Review. Endocr. Pract..

[93] Drincic A.T., Armas L.A., van Diest E.E., Heaney R.P. (2012). Volumetric Dilution, Rather Than Sequestration Best Explains the Low Vitamin D Status of Obesity. Obesity.

[94] Plebani M., Zaninotto M., Giannini S., Sella S., Fusaro M., Tripepi G., Gallieni M., Herrmann M., Cozzolino M. (2024). Vitamin D assay and supplementation: Still debatable issues. Diagnosis.

[95] Tang J.C., Dunn R., Dutton J.J., Farag A., Piec I., Chipchase A., Greeves J., Fraser W.D., Webb E.A. (2024). Measurement of 1,25-dihydroxyvitamin D in serum by LC-MS/MS compared to immunoassay reveals inconsistent agreement in paediatric samples. Clin. Chem. Lab. Med..

[96] Bassatne A., Chakhtoura M., Saad R., Fuleihan G.E.-H. (2019). Vitamin D supplementation in obesity and during weight loss: A review of randomized controlled trials. Metabolism.

[97] Sarathi V., Karethimmaiah H., Goel A. (2017). High-dose Vitamin D supplementation precipitating hypercalcemic crisis in granulomatous disorders. Indian J. Endocrinol. Metab..

[98] Tannous P., Fiscaletti M., Wood N., Gunasekera H., Zurynski Y., Biggin A., Kilo T., Hayes E., Munns C. (2019). Safety and effectiveness of stoss therapy in children with vitamin D deficiency. J. Paediatr. Child Health.

[99] O’dOnnell J.E.M., Leach S.T., Bowcock N.L., Chen S., Gupta N., Jiang K., Lopez R.N., Messenger R., Nahidi L., Shapiro A. (2025). Daily Vitamin D3 Versus Stoss Vitamin D3 for Correction of 25OHD Deficiency in Children with Inflammatory Bowel Disease, a Randomised Controlled Trial. Dig. Dis. Sci..

[100] Radicioni M., Caverzasio C., Rovati S., Giori A.M., Cupone I., Marra F., Mautone G. (2022). Comparative Bioavailability Study of a New Vitamin D3 Orodispersible Film Versus a Marketed Oral Solution in Healthy Volunteers. Clin. Drug Investig..

[101] Kanagalingam T., Khan T., Sultan N., Cowan A., Thain J., Hoy C., Ledger S., Clemens K.K. (2023). Reducing the risk of denosumab-induced hypocalcemia in patients with advanced chronic kidney disease: A quality improvement initiative. Arch. Osteoporos..

[102] Jim H.S.L., Quinn G.P., Gwede C.K., Cases M.G., Barata A., Cessna J., Christie J., Gonzalez L., Koskan A., Pidala J. (2013). Patient education in allogeneic hematopoietic cell transplant: What patients wish they had known about quality of life. Bone Marrow Transplant..

[103] Courbebaisse M., Bourmaud A., Souberbielle J.-C., Sberro-Soussan R., Moal V., Le Meur Y., Kamar N., Albano L., Thierry A., Dantal J. (2023). Nonskeletal and skeletal effects of high doses versus low doses of vitamin D3 in renal transplant recipients: Results of the VITALE (VITamin D supplementation in renAL transplant recipients) study, a randomized clinical trial. Am. J. Transplant..

[104] Kharfan-Dabaja M.A., Aljurf M. (2017). Hematopoietic cell transplantation: Training challenges and potential opportunities through networking and integration of modern technologies to the practice setting. Hematol. Stem Cell Ther..

[105] Davidson Z.E., Walker K.Z., Truby H. (2012). Do Glucocorticosteroids Alter Vitamin D Status? A Systematic Review with Meta-Analyses of Observational Studies. J. Clin. Endocrinol. Metab..

[106] Bar M., Ott S.M., Lewiecki E.M., Sarafoglou K., Wu J.Y., Thompson M.J., Vaux J.J., Dean D.R., Saag K.G., Hashmi S.K. (2020). Bone Health Management After Hematopoietic Cell Transplantation: An Expert Panel Opinion from the American Society for Transplantation and Cellular Therapy. Biol. Blood Marrow Transplant..

[107] Jackmann N., Mäkitie O., Harila-Saari A., Gustafsson J., Dernroth D.N., Frisk P. (2020). Vitamin D status in children with leukemia, its predictors, and association with outcome. Pediatr. Blood Cancer.

[108] Alexandru A., Ivan C.-S., Tanasescu S., Oprisoni L.A., Dragomir T.-L., Varga N.-I., Mateescu D., Diaconu M., Margan M.-M., Boeriu E. (2024). Are Pediatric Cancer Patients a Risk Group for Vitamin D Deficiency? A Systematic Review. Cancers.

[109] Yalniz F.F., Murad M.H., Lee S.J., Pavletic S.Z., Khera N., Shah N.D., Hashmi S.K. (2018). Steroid Refractory Chronic Graft-Versus-Host Disease: Cost-Effectiveness Analysis. Biol. Blood Marrow Transplant..

[110] Kim N.V., McErlean G., Yu S., Kerridge I., Greenwood M., Lourenco R.D.A. (2025). Healthcare Resource Use and Costs of Allogeneic Hematopoietic Stem Cell Transplantation Complications: A Scoping Review. Curr. Oncol..

[111] Carrillo-Cruz E., García-Lozano J.R., Márquez-Malaver F.J., Sánchez-Guijo F.M., Cuadrado I.M., i Coll C.F., Valcárcel D., López-Godino O., Cuesta M., Parody R. (2019). Vitamin D Modifies the Incidence of Graft-versus-Host Disease after Allogeneic Stem Cell Transplantation Depending on the Vitamin D Receptor (VDR) Polymorphisms. Clin. Cancer Res..

[112] Broers A.E.C., de Jong C.N., Bakunina K., Hazenberg M.D., Kooy M.v.M., de Groot M.R., van Gelder M., Kuball J., van der Holt B., Meijer E. (2022). Posttransplant cyclophosphamide for prevention of graft-versus-host disease: Results of the prospective randomized HOVON-96 trial. Blood Adv..

[113] Hudda Z., Davies S.M., Lane A., Schiff D.E., Anderson E.J., Mehta P.A., Myers K.C., Gloude N.J. (2025). Abatacept Prevents Severe Acute Graft-Versus-Host Disease Without Increasing Graft Failure Risk in Pediatric Bone Marrow Failure Syndromes. Pediatr. Blood Cancer.

[114] Iqbal M., Nieto F.A.M., Brannick K.M., Li Z., Murthy H., Foran J., Roy V., Kharfan-Dabaja M.A., Ayala E. (2023). A Calcineurin Inhibitor Free Graft Versus Host Disease Prophylaxis for Patients Undergoing Matched Related and Matched Unrelated Donor Allogeneic Hematopoietic Cell Transplant. Biol. Blood Marrow Transplant..

[115] Wierzbicka A., Oczkowicz M. (2022). Sex differences in vitamin D metabolism, serum levels and action. Br. J. Nutr..

[116] Weich L., Brummer C., Ghimire S., Peter K., Althammer M., Babl N., Voll F., Bruss C., Hoering M., Wallner S. (2025). Impact of Liver and Kidney Function on Vitamin D3 Metabolism in Female and Male Patients Undergoing Allogeneic Hematopoietic Stem-Cell Transplantation. Int. J. Mol. Sci..

[117] Dupuis M.L., Pagano M.T., Pierdominici M., Ortona E. (2021). The role of vitamin D in autoimmune diseases: Could sex make the difference?. Biol. Sex Differ..

[118] Caserta S., Gangemi S., Murdaca G., Allegra A. (2023). Gender Differences and miRNAs Expression in Cancer: Implications on Prognosis and Susceptibility. Int. J. Mol. Sci..

[119] Allegra A., Caserta S., Genovese S., Pioggia G., Gangemi S. (2023). Gender Differences in Oxidative Stress in Relation to Cancer Susceptibility and Survival. Antioxidants.

